# COVID-19 and Comorbidities: What Has Been Unveiled by Metabolomics?

**DOI:** 10.3390/metabo14040195

**Published:** 2024-03-30

**Authors:** André Luiz Melo Camelo, Hans Rolando Zamora Obando, Isabela Rocha, Aline Cristina Dias, Alessandra de Sousa Mesquita, Ana Valéria Colnaghi Simionato

**Affiliations:** 1 Laboratory of Analysis of Biomolecules Tiselius, Department of Analytical Chemistry, Institute of Chemistry, Universidade Estadual de Campinas (UNICAMP), Campinas 13083-970, São Paulo, Brazil; a266017@dac.unicamp.br (A.L.M.C.); h230022@dac.unicamp.br (H.R.Z.O.); i218142@dac.unicamp.br (I.R.); a203710@dac.unicamp.br (A.C.D.); a193440@dac.unicamp.br (A.d.S.M.); 2 National Institute of Science and Technology for Bioanalytics—INCTBio, Institute of Chemistry, Universidade Estadual de (UNICAMP), Campinas 13083-970, São Paulo, Brazil

**Keywords:** metabolomics, SARS-CoV-2, COVID-19, comorbidities

## Abstract

The COVID-19 pandemic has brought about diverse impacts on the global population. Individuals with comorbidities were more susceptible to the severe symptoms caused by the virus. Within the crisis scenario, metabolomics represents a potential area of science capable of providing relevant information for understanding the metabolic pathways associated with the intricate interaction between the viral disease and previous comorbidities. This work aims to provide a comprehensive description of the scientific production pertaining to metabolomics within the specific context of COVID-19 and comorbidities, while highlighting promising areas for exploration by those interested in the subject. In this review, we highlighted the studies of metabolomics that indicated a variety of metabolites associated with comorbidities and COVID-19. Furthermore, we observed that the understanding of the metabolic processes involved between comorbidities and COVID-19 is limited due to the urgent need to report disease outcomes in individuals with comorbidities. The overlap of two or more comorbidities associated with the severity of COVID-19 hinders the comprehension of the significance of each condition. Most identified studies are observational, with a restricted number of patients, due to challenges in sample collection amidst the emergent situation.

## 1. Introduction

Severe Acute Respiratory Syndrome Coronavirus 2 (SARS-CoV-2) is the pathological agent accountable for the respiratory disease known as Coronavirus Disease 2019 (COVID-19) [1]. In December 2019, in the city of Wuhan, China, the first cases were reported. The virus quickly spread worldwide, being declared a pandemic by the World Health Organization (WHO) on 11 March 2020, leading to nearly 7 million deaths worldwide [2]. Fever, shortness of breath, cough, and loss of smell and taste are common symptoms of COVID-19 [3]. The disease can be manifested in asymptomatic cases up to severe cases, which require hospitalization and intensive care [4,5].

**Figure 1:**
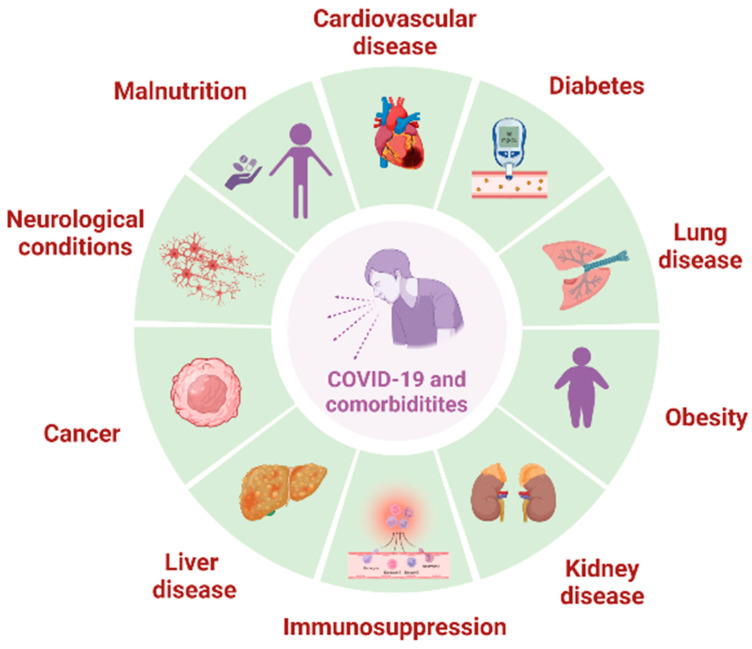
The most common comorbidities associated with severe COVID-19.

The presence of pre-existing conditions in patients can increase the risk of COVID-19 complications [6]. Figure 1 illustrates the comorbidities most commonly associated with severe COVID-19, including cardiovascular diseases, diabetes, lung disease, obesity, and kidney disease [7,8,9,10,11]. Moreover, other comorbidities such as cancer, liver disease, immunosuppression, neurological conditions, and malnutrition may influence increased complications of COVID-19 [12,13,14,15,16]. Despite vaccination of the population has provided significant progress in reducing COVID-19 cases and WHO’s declaration about the end of the global disease emergency, many unanswered inquiries about the metabolic correlation of comorbidities and COVID-19 still remain.

Metabolomics is a field of study that focuses on the comprehensive analysis of metabolites of an organism or biological system [17]. It plays an important role in understanding metabolic changes associated with disease and represents a potential area for studying and understanding the influence of comorbidities on SARS-CoV-2 infection [18]. Moreover, metabolomics can assist the researcher in identifying therapeutic targets and understanding the metabolic pathways affected by the presence of comorbidities in COVID-19 [19].

Metabolomics is considered a multidisciplinary field that employs advanced technology for appropriate workflow [20]. Analytical techniques such as chromatography, capillary electrophoresis, mass spectrometry, and nuclear magnetic resonance have often been used for this purpose [21,22,23]. Moreover, machine learning tools and databases have become increasingly important in the qualitative and quantitative analysis of metabolites data, especially when dealing with complex and varied datasets obtained from analytical platforms [24,25].

These integrated approaches enable a more comprehensive understanding of metabolic profiles and their relationship to disease, comorbidities, and severity, providing valuable information for research and development of personalized therapies [26]. Although metabolomics has significant application potential, there are few studies focusing specifically on the presence of coexisting diseases and COVID-19 using a metabolomics approach.

In this context, applying metabolomics to the study of COVID-19 and previous comorbidities represents a promising avenue with the potential to provide important insights into the metabolic profiles associated with these conditions and may contribute to the development of more accurate and effective diagnostic strategies, patient stratification, and therapeutic approaches [27,28,29].

Therefore, this article aims to critically compile the scientific production related to metabolomics in the specific context of COVID-19 and comorbidities. For an adequate organization, the topics were divided according to the most prevailing comorbidities. Thus, this article presents to researchers, and to those interested in this subject, a fragmented view of existing publications. It also presents the potential areas to be explored, allowing a deeper comprehension of the metabolic interaction between pre-existing diseases and COVID-19, as well as the associated outcomes, and their long-term impact on the lives of infected individuals.

## 2. Methods

**Figure 2:**
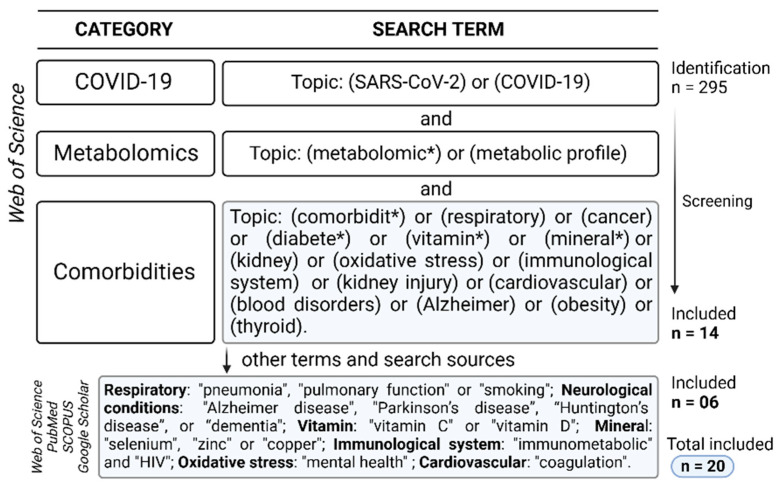
Scheme of the study selection process. * Boolean operator.

The research involved the use of the Web of Science database as a search tool for identifying potential studies aligned with the objectives of this investigation. The scope of consideration included works published from 1 January 2020, to 31 May 2023. The topic “field” of the Web of Science was employed, and only articles were included. Initially, three levels of searches were conducted simultaneously to encompass the principal terms involved in the categories: (i) COVID-19; (ii) metabolomics; and (iii) comorbidities. Subsequently, given the limited availability of research pertaining to certain comorbidities, a subcategory was incorporated into the searches to specifically cover pathologies associated with the primary term of the comorbidity. Furthermore, additional databases (PubMed, SCOPUS, and Google Scholar) were also explored using specific terms. The selection criteria primarily considered studies that involved metabolomics research related to COVID-19 and any type of comorbidity. Figure 2 shows the complete set of criteria used in the research.

## 3. Results of the Selection Process

Twenty metabolomic studies reporting a range of metabolites associated with comorbidities and COVID-19 were identified. Most studies employed a targeted approach using flow injection analysis coupled to tandem mass spectrometry (FIA-MS/MS) and ultra high-performance liquid chromatography–high-resolution mass spectrometry (UHPLC-HRMS) techniques. Analysis of the studies revealed commonly altered metabolic pathways, such as the kynurenine pathway, polyunsaturated fatty acid metabolism, and amino acid metabolism, providing significant insights into the metabolic impact of COVID-19 on patients with pre-existing conditions. The Supplementary Material (Table S1) shows detailed information, which includes data on the identified metabolites, the analytical platforms, the metabolomic approaches, and the observed metabolic interactions, thereby enhancing the development of targeted therapeutic strategies. Surprisingly, literature searches did not include studies directly addressing Alzheimer’s disease and malnutrition alongside COVID-19 within the metabolomics context. Nonetheless, we highlighted the importance of these two comorbidities, since investigating their metabolic interactions with COVID-19 may uncover new perspectives for understanding and treating these conditions in future research. Complete references of primary and secondary articles are available for consulting, providing a basis for future investigations.

### 3.1. Diabetes

Diabetes is a serious and common chronic disease, possibly causing fatal complications, disability, and a reduction of life expectancy [30,31]. It represents a metabolic dysfunction marked by elevated blood glucose levels resulting from inadequate insulin production by pancreatic β-cells [32]. Diabetes has several forms, but the most common one is type 2 diabetes (T2D), and it usually occurs in adults [31,33]. According to WHO, approximately 422 million individuals grapple with diabetes globally, with most residing in low- and middle-income nations [33]. Annually, diabetes directly contributes to 1.5 million deaths. The incidence and prevalence of diabetes have shown a consistent upward trend over the past few decades [33]. It is estimated that by 2045, the number of subjects with diabetes in the world will be 600 million [31].

The scientific literature asserts that diabetes is a risk factor for other diseases, e.g., cardiovascular [34,35], kidney [35], and immunosuppressive diseases [36], and respiratory tract infections [32,37]. The harmed immune system—together with the metabolic disorder—increases the susceptibility of patients to various pathogens, including SARS-CoV-2 [38]. In this case, innate immune pathways play an important role in the defense of cells against pathogenic infection [39]. Invasive viruses are identified by a pattern recognition receptor (PRR), triggering the expression of type I interferons (IFN) that manifest antiviral effects [40]. Besides interfering with IFN, the immune defects associated with diabetes mellitus can prevent the production of interleukin-22 (IL-22), which reduces chronic inflammation, eliciting antimicrobial immunity, maintaining the integrity of the intestinal mucosal barrier, and enhancing insulin sensitivity [41,42,43].

Given the conditions diabetes mellitus imposes on the immune system, viral pathogens find favorable conditions in patients with diabetes to replicate and survive. Several observational and review studies have been published reporting the complications and outcomes of patients with comorbidities infected by SARS-CoV-2 [44,45]. An observational study of patients admitted to the intensive care unit (ICU) at Wuhan Jin Yin-tan hospital (Wuhan, China) from late December 2019 to 26 January 2020 revealed that of the nine diabetic patients with COVID-19 admitted to the unit during the period, only two survived [46]. Another observational study assessing a larger cohort composed of clinical records in a database (n = 17,278,392) pointed out that diabetes was a risk factor for COVID-19, resulting in 10,926 obits [47]. However, the presence of other factors alongside diabetes contributed to this tragic outcome, precluding the attribution of deaths solely to the interaction between the virus and diabetes [47].

Although the general understanding about the association between diabetes and COVID-19 has improved, many uncertainties still remain, and such observations suggest the existence of complex factors associated with them. The first metabolomics research involving diabetic patients with COVID-19, also considered hypertension and was published in early 2022 [48]. In this study, 115 patients with COVID-19 were classified according to disease severity, hypertension status, and diabetes status. Blood samples from patients were subjected to targeted metabolomics analysis using FIA-MS/MS and liquid chromatography coupled to tandem mass spectrometry (LC-MS/MS). An increase in disease severity among diabetic patients correlated with a reduction in triacylglycerol levels, including docosapentaenoic (C22:5, DPA), palmitic (C16:0), and docosahexaenoic (C22:6, DHA) acids (false discovery rate—FDR < 0.01). Functional enrichment analysis indicated that the triacylglycerols pathway was significantly affected in individuals with severe COVID-19 and diabetes (FDR = 7.1 × 10^−27^) [48].

**Figure 3:**
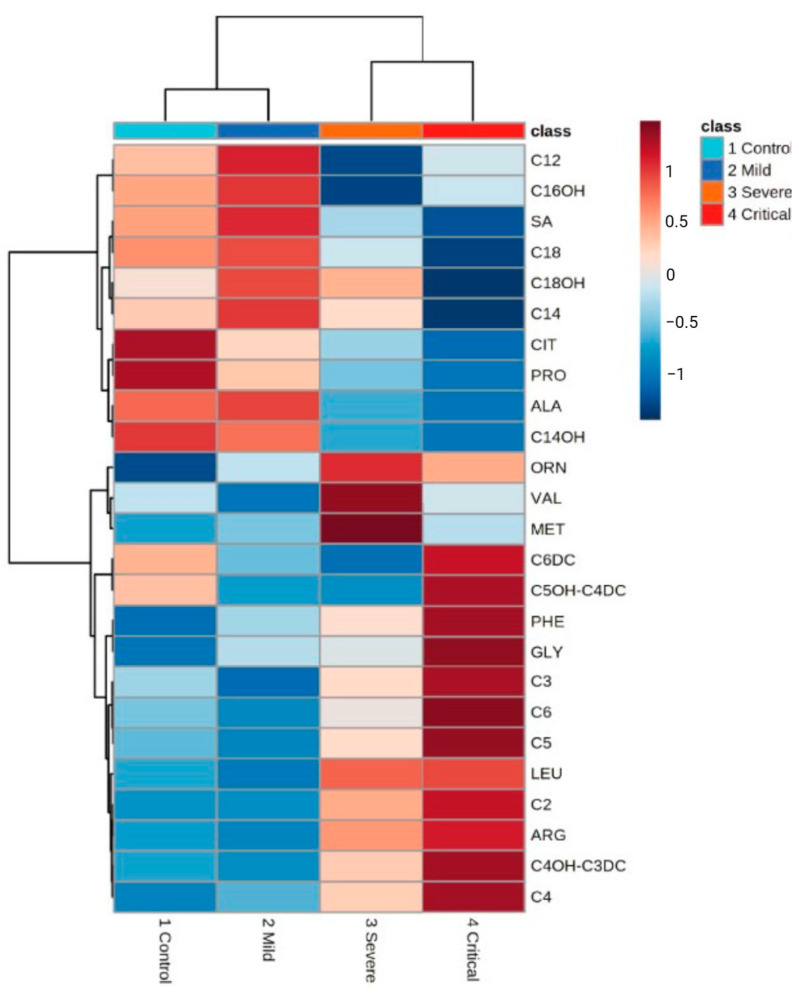
Hierarchical clustering analysis to evaluate variations in the abundance of metabolites across the study groups. Link to the license, accessed on 14 March 2024, (https://creativecommons.org/licenses/by/4.0/deed.en). Image credit and adapted from Martínez-Gomez et al. [49].

A study conducted by Martínez-Gomez et al. aimed to analyze the metabolome of healthy individuals and patients exhibiting mild, severe, and critical symptoms of COVID-19 severity [49]. The participants were divided into four distinct groups. The percentages of individuals with T2D in the severe symptoms group (n = 60 = 35.0%) and critical symptoms groups (n = 210 = 35.2%) were higher compared with the healthy (n = 31 = 9.6%) and the mild symptoms groups (n = 152 = 8.5%). Other clinical characteristics, such as hypertension and obesity, were considered in the classification. Hierarchical clustering analysis (Figure 3) showed variations in the intensity of metabolites within the examined groups. This highlights that concentration of certain metabolites differs as the severity of the disease increases. Succinylacetone, citrulline, proline, alanine, and phenylalanine were identified as the main metabolites contributing to the variability in each group, with phenylalanine emerging as a potential biomarker for disease severity under the evaluated conditions [49]. Despite the significant contribution of the study, it holds some limitations, such as the experimental design that did not allow for the monitoring of metabolites throughout the disease progression, and the disregard on specific comorbidities. Therefore, prospective studies are needed to provide a more comprehensive characterization of the metabolome as the disease advances and to better understand its specific association with diabetes.

Maltais-Payette et al. employed a targeted metabolomics approach to explore the association between severe COVID-19 symptoms and the concentrations of circulating amino acids, including leucine, isoleucine, valine, glutamate, phenylalanine, proline, glutamine, cysteine, and alanine [50]. The authors used LC-MS/MS to quantify these metabolites in patients’ plasma, including diabetic and obese patients with severe and critical symptoms. A significant and positive correlation between the severity of COVID-19 symptoms and the presence of phenylalanine was revealed. This result aligns with a previous study by Martínez-Gomez et al., who also observed a relationship between the presence of phenylalanine and severe COVID-19 symptoms [49]. However, the authors attributed the presence of phenylalanine to obesity, and emphasized that, in addition to the impact of high adiposity, the severity of COVID-19 also impacts on amino acid metabolism. This suggests that phenylalanine significantly contributes to the severity of the disease symptoms. These findings support previous discoveries and indicate that severe COVID-19 and the presence of comorbidities affect amino acid metabolism [50].

In the initial phases of COVID-19 vaccine development, countries implementing neonatal Bacillus Calmette–Guérin (BCG) vaccination had fewer cases and lower mortality rates of COVID-19 compared with countries without such vaccination, sparking researchers’ exploration of the potential cross-protective effects of the BCG vaccine against COVID-19 [51,52]. To assess the efficacy of BCG against COVID-19 in diabetic patients, a retrospective study was conducted by Anwardeen et al. using serum samples from 77 COVID-19 patients stratified into diabetic and non-diabetic groups, based on their previous BCG vaccination status [53]. These samples were subjected to targeted metabolomic analysis. The analysis of the metabolic profile in BCG-vaccinated patients with T2D, compared with non-vaccinated patients, demonstrated enrichment of specific metabolites in the former group. These included sarcosine, aconitic acid, and cholesterol esters (CE 20:0, 20:1, 22:2). On the other hand, spermidine and glycosylceramides (Hex3Cer(d18:1_22:0), Hex2Cer(d18:1/22:0), HexCer(d18:1/26:1), Hex2Cer(d18:1/24:0), HexCer(d18:1/22:0)) were found at higher concentrations in non-BCG-vaccinated patients. Moreover, the data suggested a reduction in sarcosine synthesis from glycine and choline, alongside an elevation in spermidine synthesis in the BCG-vaccinated cohort, observed in T2D and non-T2D groups, respectively. Additionally, the research highlights the potential negative effect of BCG in diabetic patients with COVID-19 [53]. Despite the relevance of the study, it has gaps that need to be addressed by future research, including a larger number of participants, sex matching, and consideration of the interval between BCG revaccination and SARS-CoV-2 infection. Furthermore, it has no conclusive data regarding the beneficial effect of the BCG vaccine against COVID-19.

Tong et al. have observed that 1,5-anhydro-D-glucitol (1,5-AG) can block viral infections at physiological concentrations in cellular models [54]. The authors demonstrate how 1,5-AG fuses the virus with the cell membrane by attaching to the S2 subunit of the spike protein of SARS-CoV-2 [54]. This antiviral effect has also been documented for other coronaviruses, including MERS-CoV. Additionally, the study highlights that the levels of this antiviral metabolite were markedly reduced in individuals with diabetes compared with those without diabetes. Administering this metabolite to diabetic mice reduced the pathological consequences induced by COVID-19, implying that 1,5-AG may represent a potential therapeutic avenue for diabetic patients [54]. Based on these findings, potential avenues for further investigation include the following: (i) the need for clinical trials to assess the administration of this metabolite in patients with diabetes, (ii) expansion of the sample cohort, and (iii) assessment of the role of 1,5-AG and other metabolites as biomarkers for discerning disease outcomes and predicting patient prognosis.

The metabolomics results involving diabetic patients infected with SARS-CoV-2 are scarce and sometimes contradictory due to the heterogeneity of the studied populations and the many limitations. The main challenge to write about this topic was finding studies focused solely on patients with diabetes. Nevertheless, it has been noted that, in general, the disease is closely related to other comorbidities commonly associated with COVID-19, such as obesity and hypertension.

### 3.2. Obesity

Obesity is a systemic chronic disease characterized by an unhealthy and disproportional increase in body weight, as a function of height, associated with an accumulation of fat. Currently, this disorder is considered a pandemic that affects adults and children of all genders. According to the WHO, overweight people are those with a body mass index (BMI) between 25 and 30, whereas those with a BMI above 30 are considered obese. Up to 2017, 4 million people died per year due to conditions related to overweight. The prevalence of obesity in children and adolescents aged 5–19 years rose from 4% to 18% from 1975 to 2019 [55,56,57].

Obesity and overweight are risk factors for noncommunicable diseases (prostate, breast, liver, colon cancers, etc.), cardiovascular diseases, metabolic syndrome, and T2D. In this respect, research has demonstrated that metabolomics is an important tool to deep our knowledge on the metabolic characteristics and changes caused by overweight and obesity [55,56,58].

Pregnancy, dietary interventions, and weight loss are some of the fronts where metabolomics has furnished a deeper insight into the altered metabolic pathway and patterns due to obesity and overweight [58]. Notable examples include increased levels of uric acid and decreased levels of 2-ketoglucose dimethyl acetal and aminomalonate which have been observed in women with overweight/obesity. Elevated concentrations of long-chain fatty acids (LCFAs) in newborns were identified as potential predictors of obesity in their later life [59]. Higher concentrations of branched-chain amino acids (BCAAs), phosphatidylcholines, triglycerides, and cholesteryl esters were found to discriminate children with obesity from those with normal weight [60]. BCAAs, lysoglycerophospolipids, glycine, and glutamate are associated with insulin resistance [61,62].

As observed in various systematic reviews and meta-analyses of the scientific literature, obesity is associated with an increased risk of worsening symptoms of COVID-19 symptoms [56,63,64,65,66]. Obesity is related to other comorbidities such as hypertension, prediabetes, dyslipidemia, kidney disease, cardiovascular disease, and respiratory dysfunctions. Therefore, it tends to increase the risk of severe COVID-19. Deng et al. showed that 32% of COVID-19 patients had obesity, putting them at a 1.79 times increased risk of 1.79 times, requiring invasive mechanical ventilation (IMV) and admission to the intensive care unit (ICU). However, the same association was not observed regarding mortality [63]. On the other hand, the meta-analysis conducted by Singh et al. suggests that obesity was associated with a high mortality among COVID-19 patients [64].

Obesity conditions have already been reported as possibly leading to a severe COVID-19 stage. Obesity is related to chronic inflammation and inflammatory makers such as IL-6 and tumor necrosis factor-α (TNF-α) [67], which may induce an overreaction of the inflammatory process in response to infection. Furthermore, obesity may increase the expression of transmembrane protease, serine 2 (TMPRSS2), and angiotensin-converting enzyme 2 (ACE2) in the lower respiratory tract, facilitating the infection and respiratory complications [68,69]. Moreover, obesity is related to respiratory diseases and a reduced pulmonary function [70].

Searching for the molecular relationship between obesity/overweight and COVID-19 severity based on a metabolomics approach in the Web of Science database (Accessed 14 June 2023) yielded only three papers, which are briefly described below.

Marín-Corral et al. assessed the critical metabolic pathways altered in severe infections of COVID-19 infections. The targeted metabolomic study was developed in plasma samples of 49 patients stratified in three subgroups according to COVID-19 severity (moderate, severe, or critical) and weight conditions (obese and non-obese groups). Ceramide metabolism, tryptophan, and nicotinamide adenine nucleotide metabolic consumption reactions were altered in both severe COVID-19 and obesity conditions. The study discovered that the critical COVID-19 group had a higher frequency of obesity than the moderate and severe groups, in accordance with the findings of the aforementioned meta-analyses [56,63,64,65,66]. Plasma ceramide (especially C18:0, C16:0, and C24:1) levels were increased with disease severity whereas hexosylceramides (HexCer 20:0, HexCer C22:0, HexCer C24:0 and HexCer C24:1) levels decreased. The alteration of the kynurenine pathway was identified due to a decrease in tryptophan concentrations and an increase in kynurenine and 3-hydroxikynurenine. Additionally, a relation between the tryptophan metabolism markers and the inflammatory markers was demonstrated. Alteration in conversion of cortisol into cortisone, α-ketoglutarate into succinate, and pyruvate into lactate indicate an unbalance of NAD+/NADH that limit the macrophage immune function in the inflammatory process [19].

Rössler et al. screened the metabolic, lipidic, and lipoprotein serum profiles of infected patients with mild-to-moderate courses (ambulatory and hospitalized) and healthy controls, by applying an in vitro diagnostic research-only (IVDr) system with nuclear magnetic resonance (NMR) analysis. They also combined those profiles to pre-selected cytokine and chemokine levels to correlate them to the records from another study, where an ambulatory care model was tested. Higher levels of phenylalanine, glutamic acid, dimethylglycine, sarcosine, ketone bodies, and creatine were found in COVID-19 patients compared with healthy ones. On the other hand, lower levels of lysine, histidine, glutamine, ornithine, isoleucine, and trimethylamine-N-oxide were found upon COVID-19 infection. Furthermore, the study showed that the ratio between the NMR inflammatory markers glycoprotein A and B (Glyc) and supramolecular phospholipid composite (SPC) has a prognostic value, since the Glyc/SPC ratio was higher in hospitalized patients than in outpatients. Higher BMIs were associated with a higher level of Glyc and lower level of SPC; however, reference values for different BMI values, gender, and age could not be stablished. Despite these interesting findings, the BMI information was used only to assess the susceptibility of those NMR markers to BMI as a potential confounder. No additional comparison between the metabolic or lipid profile considering obese/overweight vs. normal-weight subgroups was explored [71].

**Figure 4:**
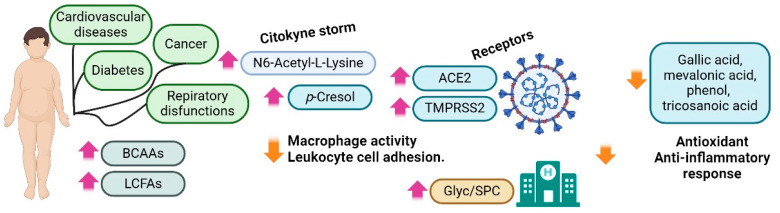
Obesity is related to other comorbidities associated with worse symptoms and prognostics of COVID-19. The purple and orange arrows indicate increase and decrease, respectively. Branched-chain amino acids (BCAAs) and long-chain fatty acids (LCFAs) are markers associated with obesity and overweight in children. In SARS-CoV-2 infection, an increased level of p-cresol is associated with an increased level of receptors angiotensin-converting enzyme 2 (ACE2) and transmembrane protease, serine 2 (TMPRSS2). The p-cresol reduces macrophage activity and leukocyte cell adhesion. The glycoprotein/supramolecular phospholipid composite ratio might be a potential prognostic marker. Some metabolites such as gallic acid and tricosanoic acid have downregulated levels, indicating a reduced anti-inflammatory response.

In a metabolomic analysis, Jalaleddine et al. directly contrasted the metabolomic profiles of lean and obese/overweight COVID-19 patients with those of lean and obese/overweight non-COVID-19 patients. Higher levels of N6-acetyl-L-lysine and p-cresol were found in the obese/overweight group. Based on the cited literature, both compounds have a role in the “cytokine storm” in response to SARS-CoV-2. For example, p-cresol increases the levels of ACE2 and TMPRSS2, which are receptors for SARS-CoV-2, and inhibits leukocyte cell adhesion and macrophage activity. Other metabolites were in lower concentrations in the obese/overweight group compared with the normal-weight group, such as gallic acid, mevalonic acid, homo-L-arginine, 3,4-dihydroxymandelic acid, phenol, and tricosanoic acid, which have a role in antioxidant and anti-inflammatory processes (Figure 4) [72].

Overall, as recent studies have evinced, obesity/overweight is an important risk factor associated with worse prognosis in SARS-CoV-2-infected patients due to connection with other COVID-19 risk factors, such as chronic conditions (pulmonary impairment, T2D, and cardiovascular diseases). Metabolomics has been used as a valuable approach to understand and obtain insight into the metabolic system alterations due to obesity/overweight. To date, scarce metabolomics’ studies have focused on investigating the specific relationship between the severity of clinical manifestations of COVID-19 and obesity. Limited accessibility to patients or complete clinical data might represent a barrier to perform thorough retrospective research. In this case, researchers should be oriented to understand the clinical and metabolomic role of obesity in patients who survived and are experiencing some post-infection sequels.

### 3.3. Cancer

Individuals with cancer are at a higher risk of experiencing severe COVID-19 compared with those without an oncological condition [73]. Common risk factors include age, smoking history, pre-existing conditions, and the stage of cancer. Lung cancer is within the main types of cancer that can aggravate the symptoms caused by SARS-CoV-2 infection [73]. In addition to the disease, treatment and post-cancer conditions can affect internal organs and make the immune system weak to healing infections [6].

**Figure 5:**
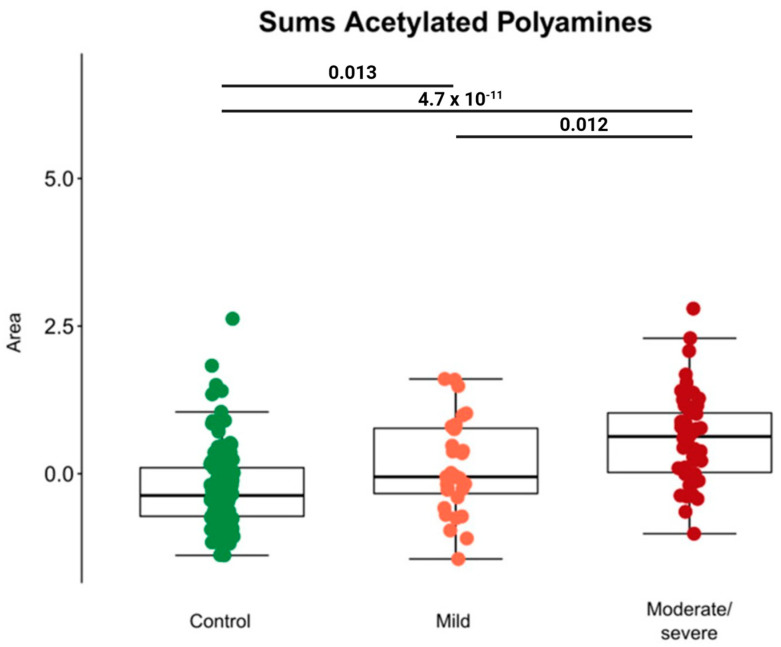
Boxplot of acetylated polyamine derivatives present among cancer patients at different levels of COVID-19 severity. Link to the license, accessed on 14 March 2024 (https://creativecommons.org/licenses/by/3.0/deed.en). Image credit to Bourgin et al. [74].

In research conducted during the COVID-19 pandemic, the serum metabolome of 204 cancer patients was investigated using a metabolomics approach. Three groups were evaluated: (i) control group—patients without a COVID-19 diagnosis and without signs of respiratory infection (n = 128); (ii) COVID-19 patients confirmed by PCR testing with mild symptoms—without invasive treatment (n = 37); (iii) COVID-19 patients confirmed by PCR testing with moderate/severe symptoms, who required respiratory assistance (n = 39). The presence or absence of other comorbidities (chronic obstructive pulmonary disease, obesity, hypertension, congestive heart failure, and diabetes mellitus), type of malignancy, cancer metastasis, and whether the patient died were also considered. The use of chromatographic and mass spectrometry techniques in the analysis of the patients’ blood serum as well as uni- and multivariate data analysis tools showed a positive correlation between the levels of circulating acetylated polyamines and the severity of COVID-19 in cancer patients [74]. They calculated the total of normalized peak areas associated with N1, N12-diacetylspermine, N1-acetylputrescin, N1, N8-diacetylspermidine, and N1-acetylspermidine for each patient. The results were presented in a boxplot, with black bars indicating *p*-values (Figure 5). The types of cancer included in the study were not specified, since the main focus was to establish the relationship between levels of acetylated polyamines and the severity of COVID-19 in oncologic patients, regardless of the specific type of cancer.

L-glutamine is the most abundant amino acid circulating in the blood and it is often released from skeletal muscles to various tissues of the body [75]. Cells that have a high division rate, such as cancer cells, require glutamine for nucleotide synthesis. Also, growing cells use glutamine to supply the mitochondria with energy by anaplerosis, which is a process of replacing intermediates in the tricarboxylic acid cycle [76]. Although most tissues can synthesize glutamine, the levels of this amino acid in membranes become critical when the organism undergoes stress [75]. Cancer cells establish a more intense metabolic demand and utilize glutamine metabolism by the anaplerosis of the tricarboxylic acid cycle to synthesize most of the non-essential amino acids that are incorporated into proteins [76].

Indeed, patients with COVID-19 and cancer have shown significantly lower levels of glutamine compared with those without the comorbidity, and deficiency of this amino acid has been associated with increased disease severity [77]. However, glutamine levels in this COVID-19 risk group are poorly explored in quantitative terms and need to be adequately delineated. For this purpose, targeted metabolomics is one of the promising approaches to identify and possibly treat glutamine deficiency in order to comprehend the role of this amino acid for the management and prognosis of these patients, helping to improve clinical outcomes and reduce COVID-19 severity.

Cancer and COVID-19 share some inflammatory characteristics. A notable feature is the elevated levels of pro-inflammatory cytokines, termed a ”cytokine storm”, that has the potential to trigger multiple organ failure [78]. Understanding the main cytokine networks involved in this process represents a promising strategy for blocking the cytokine storm. Constantini et al. employed untargeted metabolomics, analyzing blood plasma, to study patients infected with SARS-CoV-2 exhibiting severe symptoms. For this purpose, blood plasma has been analyzed by proton nuclear magnetic resonance (1H-NMR). They observed that low levels of 3-hydroxybutyrate, lactate, leucine, phenylalanine, C-X-C motif chemokine ligand 9 (CXCL9), C-X-C motif chemokine ligand 10 (CXCL10), hepatocyte growth factor (HGF), interleukin-6 (IL-6), and stem cell factor (SCF) correlated with favorable patient outcomes [79]. The authors suggest that 3-hydroxybutyrate, lactate, leucine, phenylalanine, and cytokines may be related to COVID-19 pathogenesis. Based on the scientific literature, the authors state that the highlighted metabolites closely resemble those associated with the onset and progression of cancer. Based on the results, the medication employed in cancer treatment can be regarded as a therapeutic approach to prevent SARS-CoV-2 infection. Nevertheless, this investigation presents a low number of participants (n = 60) who were classified into individuals who were discharged and those who were deceased. Additionally, the number of individuals with some type of cancer (n = 4) in the study is too low for conducting a comparative study between individuals with cancer vs. with COVID-19, revealing opportunities for future research.

Denkinger et al. conducted a pilot study using convalescent plasma from individuals vaccinated against COVID-19 applied in high-risk patients [80]. A comprehensive analysis was conducted on 134 hospitalized individuals with severe COVID-19. The cohort was divided into two homogeneous groups: (i) with standard COVID-19 treatment, and (ii) with standard treatment plus convalescent plasma. Among the patients, 56 had some form of cancer (the most prevalent cancers included 20 cases of B cell lymphoma malignancies, 12 cases of acute myeloid leukemia/myelodysplastic syndrome, 11 cases of myeloma, and 9 cases of solid cancer). Two parameters were evaluated in this study: (i) clinical improvement assessed by a seven-point scale, and (ii) time to discharge and overall survival. In the overall assessment, patients who received the plasma showed no significant difference to the patients who did not receive it. However, the recovery time was shorter, and the neutralizing antibody activity was higher in cancer patients who received plasma. The study presents only the clinical outcome of patients, but leaves gaps regarding the molecular level. Additionally, the number of individuals is low, and the clinical conditions of patient admission have other non-homogeneous variables (age, sex, comorbidities, and therapy) that can influence the outcome and may not be applicable to all types of cancer. Therefore, an untargeted metabolomic approach could have been explored for the functional understanding of potential biomarkers among the investigated groups.

### 3.4. Kidney Disease

COVID-19 was firstly considered as a respiratory disease; however, increasing evidence suggests it is a complex multisystemic disease [81]. Furthermore, patients with COVID-19 often show signs of renal involvement in addition to respiratory symptoms [82]. Acute kidney injury (AKI) is defined as a rapid increase in serum creatinine, and a decreased urine output is a common problem in COVID-19, since it increases mortality, especially when patients are hospitalized in intensive care units (ICU) [83,84].

Most patients showing severe or critical course of COVID-19 have pre-existing conditions, generally hypertension, diabetes mellitus, and obesity [85]. Studies have shown that patients with chronic renal failure should also be careful about exposure to the SARS-CoV-2 virus, since chronic kidney disease (CKD) increases the risk of a severe COVID-19 course [86].

Studies specifically examining patients who developed AKI have identified several risk factors associated with its progression. These factors include advanced age, diabetes, hypertension, cardiovascular diseases, and particularly, respiratory failure [87]. An association between male sex and advanced age as key risk factors for the development of AKI has also been reported [88]. The association of AKI as one of post-COVID comorbidities, indicates the urgency of broader investigation on this topic [89].

The pathophysiology of AKI related to COVID-19 is complex, and systemic inflammation and immune response, activation of the coagulation pathway, renin—angiotensin system, endothelial injury, and other factors are involved in the renal failure process occurring in COVID-19 patients [8]. The high expression of the ACE2 receptor on tubular epithelial cells proximal to the kidneys may become a potential target for renal injury [90].

The most general reasons for AKI are surgery, septic shock, cardiogenic shock, hypovolemia, and drug toxicity [91]. The cause for AKI among individuals with COVID-19 may be multifactorial, involving a direct attack by SARS-CoV-2 on microcirculatory dysfunction, hemodynamic instability, renal congestion, microvascular thrombi, and endothelial dysfunction that are frequently found in critically ill patients [92]. Having clinical tools that allow early detection of patients at risk and with progressing AKI would be critical for attaining a positive result for the patient. Also, finding prognostic and prediction biomarkers is important to determine the progression of AKI and analyze the response to therapy, the sequential need for renal replacement therapy, and the degree of renal recovery or lingering chronic kidney ailment after AKI [93].

Technologies and computing instruments allow for a more detailed and comprehensive exploration of many metabolites in a single assessment, offering a wider perspective on disease mechanisms. Metabolomics has allowed the detailed investigation of kidney diseases by assessing related molecular pathways and has initiated the development of novel molecular diagnostic tools for nephrology [94,95].

Vergara et al. performed urine analysis of AKI/COVID-19 patients by a targeted metabolomics approach [96]. The subgroups consisted of 28 COVID-19 patients with AKI, 30 COVID-19 patients without AKI, and 24 healthy controls, which were analyzed by LC-MS. Of the 250 detected metabolites, 189 were successfully identified. The analysis revealed significant modifications in the urinary metabolic profile of COVID-19 patients compared with the controls. Among the increased metabolite levels, several essential amino acids, including phenylalanine, threonine, isoleucine, lysine, tryptophan, and leucine alongside their related metabolites, were found. Enrichment analysis indicated an association of these metabolites with amino acid metabolic pathways, particularly the kynurenine–quinolinic acid pathway of tryptophan metabolism. A connection was noted between amino acid excretion and the ACE2 level in urine. Additionally, higher levels of certain amino acid metabolites—such as indoleacetic acid and hydroxyphenylpyruvic acid—were found in the group of patients with AKI. These results provide insights into the metabolic alterations associated with COVID-19 and suggest an involvement of amino acid metabolism in the pathogenesis of the disease.

Thus, understanding the role of metabolism via viral pathogenesis is crucial, since metabolomics-based studies of emerging pathogens will help understand the metabolic changes of COVID-19 [97].

### 3.5. Cardiovascular Diseases and Blood Disorders

Cardiovascular diseases are a potential risk factor for COVID-19, since several studies show that patients with a history of hypertension and coronary heart disease have the highest mortality rate within those hospitalized with COVID-19 [98,99,100]. Cardiac complications, such as cardiac arrest, are outcomes that occur in about 3% of the patients with pneumonia [100,101]. Systemic inflammatory processes contribute to cardiac arrest in acute respiratory infections, including the release of pro-inflammatory cytokines—mediators of atherosclerosis, causing plaque rupture by local inflammation. Such infections can also induce hemodynamic and pro-coagulant effects, which predispose individuals to thrombosis and ischemia [102].

A longitudinal multi-omics study of the Indian population published in 2021 compared non-severe and severe COVID-19 patients, regardless of records on previous diseases. The integrated pathway analysis using metabolomics and proteomics revealed alterations in myeloid leukocyte activation pathway, coagulation cascade, platelet aggregation, and arginine and proline metabolism at the severe stage of COVID-19 infection [103]. Focusing on severe cases of COVID-19, Li et al. performed a longitudinal untargeted metabolomics investigation of serum samples from COVID-19 patients by UHPLC coupled to Q Exactive HFX mass spectrometer analysis. It was observed that five ornithine-related metabolites (3-amino-2-piperidone, ornithine, aspartic acid, N-acetylornithine, and asparagine) are correlated with multiple cytokine and coagulation indexes (e.g., active part thromboplastin ratio, international normalized ratio, and prothrombin time) [104].

Rizvi et al. used a hamster model to study the cardiovascular complications caused by SARS-CoV-2, observing that the initial phase of the infection leads to an acute inflammatory response and lung pathologies, whereas the late phase causes cardiovascular complications, with ventricular wall thickening associated with an increased ventricular mass/body mass ratio, and interstitial coronary fibrosis. The SARS-CoV-2 late phase was also marked by increased levels of serum cardiac troponin I, cholesterol, low-density lipoprotein, and long chain fatty acid triglycerides. The metabolomics analysis showed 62 significant metabolites in the serum samples, including 50 at higher levels and 12 at lower levels during infection [105]. This model identified N-acetylneuraminate as a potential biomarker, which has been previously associated with coronary artery disease and myocardial injury, and was also reported by other authors in the serum of COVID-19 patients [26,105,106].

According to the Center for Disease Control and Prevention (CDCP), blood disorders or hematologic malignancy includes venous thromboembolism, hemophilia, hereditary hemorrhagic telangiectasia, sickle cell disease, thalassemia, and Von Willebrand disease [107]. Abi Vijenthira et al. performed a pooled metadata-analysis study to quantify the outcomes (deaths, hospitalizations, and complications) of patients with previous hematologic malignancy that had COVID-19, concluding that adult patients have a 34% risk of death, whereas pediatric patients had a 4% risk [108]. Patients with severe or fatal COVID-19 present an increment of white blood cells count, and decrement of hemoglobin, neutrophil, lymphocyte, platelet, and eosinophil count [109]. However, to our knowledge, no metabolomics analyses to comprehend which metabolic pathways contribute to increased risks for patients with pre-existing hematologic diseases have been published, although case reports of patients with COVID-19 who had clinical outcomes of cardiac complications, as well as the cellular level responses of antibodies against SARS-CoV-2, are commonly found [110,111,112,113,114,115,116,117,118].

### 3.6. Alzheimer’s Disease

Parkinson’s disease (PD), Alzheimer’s disease (AD), and Huntington’s disease are caused by vascular disorders, infections, structural damages, and functional impairments that affect the central and/or peripheral nervous system. Particularly, AD affects more than 46 million people worldwide, as a chronic degenerative disease that accounts for approximately 60% of the dementia cases [119]. This disease is characterized by an alteration in memory, learning, behavior, and cognition. Although the causes are still unclear, accumulation of β-amyloid plaques and neurofibrillary tangles are related to the worsening of AD [120].

Among the myriad of studies involving these diseases, metabolomics analysis has aided in elucidating underlying molecular physiopathology. Researchers summarized the molecular alterations by NMR and MS-based metabolomics approaches. More specifically, studies on AD pointed out metabolites related to alterations of brain energy metabolism, such as glucogenic metabolism (glucose), and glycolytic metabolism (lactate, glutamate, and glutamine). Furthermore, metabolites related to the brain’s structure (cortisol) and integrity of the cell membranes (phospholipids, acylcarnitine, phosphatidylcholines, and phosphocholines) are recognized as biomarkers of this disease [121].

A recent review depicts the relevant role of proteomics and metabolomics in the discovery of new altered biochemical pathways in AD, PD, and amyotrophic lateral sclerosis (ALS) and how those approaches have allowed for a deeper understanding of the underlying mechanisms [122]. However, the standardization of the procedures in omics sciences to strengthen the repeatability and readability of the future studies is a challenge that must still be addressed. Thus, multi-omics research seems to be the next step for an enhanced comprehension on such diseases [122].

Patients affected by neurodegenerative diseases and dementia present a higher risk of contracting COVID-19. Clinical studies have demonstrated that infection of these patients with SARS-CoV-2 increases the disease severity leading to higher mortality rates (~40%) compared with those without dementia [123,124].

COVID-19 clinical symptoms involving neurological manifestations have been reported to affect from 15% to 88% of hospitalized cases [125,126]. Some of the most common manifestations (headache, dizziness, nausea, neuralgia, acute cerebrovascular disease, meningoencephalitis, and decreased awareness) and the less common ones (dysautonomia, Guillain–Barré syndrome, optic neuritis, and Miller Fisher syndrome) have their bases on morphological and biochemical changes to brain substructures [127]. Surprisingly, the typical symptoms (dyspnea, dysgeusia, fever, and dry cough) in patients with these pre-existing conditions are less frequent [124].

**Figure 6:**
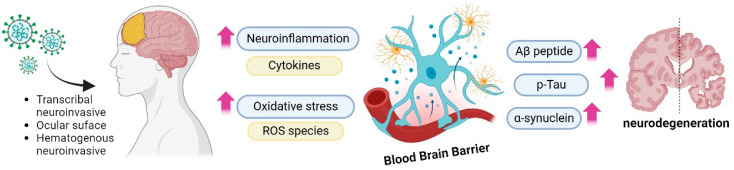
Effect of SARS-CoV-2 infection in the hyperactivation of the immune system leading to a neuroinflammatory process and oxidative stress that might weaken the blood–brain barrier facilitating the migration of peptides and proteins which accumulates in the prefrontal cortex causing neurodegenerative injuries. The purple arrows indicate an increase.

SARS-CoV-2 has impacted the central nervous system (CNS) and the sequels are still unclear (long-term COVID-19) [128]. To date, evidence points to three possible main virus entry pathways into host cells: via cranial nerves, via ocular surface, and/or via hematogenous route. In all of them, the presence and interaction with the primary receptor ACE2, neuropiline-1, and transmembrane serine protease 2 (TMPRSS2) allows the endocytosis of the virus [127]. Biochemical alterations were observed in the neuroinflammatory response and oxidative stress [127]. These processes are involved with the protein aggregation related to the neurological dysfunction associated with AD and PD, which draws attention to the importance of metabolomics studies, especially targeted metabolomics on lipid peroxidation, ROS accumulation, protein nitration, and cytokine release related pathways (Figure 6).

COVID-19 and AD biochemical mechanisms are related because both alter the blood–brain barrier homeostasis [129]. Also, SARS-CoV-2 alters the frontal cortex, a cerebral region involved with mental processes such as memory and reasoning, which is especially impaired by an advanced AD stage [130]. Chiricosta et al. investigated the transcriptome and interactome profiles of four groups: (i) patients with AD who died from COVID-19 (AD + COVID-19); (ii) patients who died of AD without COVID-19 (AD); (iii) COVID-19 patients who died without AD (COVID-19); and (iv) control individuals. The study revealed that dysregulations on several genes lead to increased neurotoxicity, higher levels of beta-amyloid, oxidative stress, and inflammation [129]. Briefly, 1664 differentially expressed genes (DEGs) were dysregulated in the controls and COVID-19 cohort, whereas 1747 DEGs were dysregulated in the AD + COVID-19 and AD cohorts. Several biological processes related to immune response were altered, including those associated with brain organization, cell cycle, oxidative stress and immune response, apoptosis and viral activity. Therefore, the authors speculate that SARS-CoV-2 increases the oxidative stress in the frontal cortex that, in turn, raises beta-amyloid toxicity [129].

Searching for metabolomics studies in Web of Science database (Accessed 30 May 2023) with the descriptors “**COVID-19**”, “**SARS-CoV-2**”, and “**metabolomics or metabolic profile**” and filtering the results by “**open access**” and “**Papers**” resulted in 142 documents. Adding the descriptor “**Alzheimer** or **Alzheimer disease**” led to only two works, but none of them referred to any research on metabolomics. The same results were obtained interchanging the descriptors to “**Parkinson’s disease**”, “**Huntington’s disease**”, or “**dementia**”. In the absence of metabolomics studies exploring the relationship of dementia diseases with SARS-CoV-2 infections, some research opportunities are open. Owing to the growing evidence that SARS-CoV-2 could increase the risk of developing AD, prospective studies should be conducted to evaluate differential markers between AD patients who never had COVID-19 and those who did [131]. Furthermore, imaging metabolomics (MALDI-TOF) may be useful to evaluate brain tissue to compare metabolic profiles between COVID-19 patients and controls [132]. Overall, further research is required to broaden our understanding on the molecular mechanisms of SARS-CoV-2 infection on dementia patients, and metabolomics is certainly an important tool in this regard.

### 3.7. Thyroid Disorders

The thyroid is a gland located in front of the trachea in the lower part of the neck [133]. It acts in the development and growth of the human body and is responsible for producing metabolism-regulator hormones [133]. Thyroid disorders are clinical conditions that affect the thyroid gland and are more frequently observed in women than in men, although the underlying causes are still unclear [134]. The most common thyroid disorders are hyperthyroidism and hypothyroidism [134], and complications associated with COVID-19 may encompass metabolic events influenced by thyroid dysfunction, which has emerged as one of the comorbidities during the pandemic [135].

Lan Wei et al. concluded that coronaviruses have effects on the thyroid glands. Autopsies showed that both parafollicular and follicular cells were damaged, justifying the low serum levels of triiodothyronine and thyroxine in patients with acute respiratory syndrome [136]. Indeed, cellular damage in COVID-19 patients can be caused by the cytokine storm that affects the thyroid glands and results in excessive inflammatory conditions, potentially exacerbating the multiple organs dysfunction, since the hormones triiodothyronine and thyroxine are associated with the functioning of brain, kidneys, liver, and heart [137,138].

Amich et al. also investigated thyroid dysfunction in COVID-19 patients divided into two pre-selected groups: (i) 334 individuals who contracted COVID-19 and (ii) 122 individuals without COVID-19 [139]. Electronic records showed that most COVID-19 patients (86.6%) were in a euthyroid state before infection, meaning their thyroid was functioning appropriately. The COVID-19 group exhibited altered levels of free thyroxine and thyroid-stimulating hormone. Furthermore, surviving patients displayed a healthy thyroid hormonal balance. The study employed a targeted approach, and the research opens opportunities for long-term investigations of other thyroid-related hormones in COVID-19 patients, as well as the possibility of an association with virus-induced sequelae [140].

COVID-19 hospitalized patients’ levothyroxine (LT4) intake has been evaluated by a metabolomics approach, comparing patients who received the hormone with euthyroid patients, namely the following: (i) patients in the ward—40 euthyroid patients and 39 patients treated with LT4; (ii) patients in the intensive care unit (ICU)—29 euthyroid patients and 9 patients treated with LT4. Baseline characteristics, alongside laboratory data, were continuously tracked, including levels of thyroid-stimulating hormone (TSH), free thyroxine (FT4), free triiodothyronine (FT3), FT3/FT4 ratio, and antiviral cytokines. Euthyroid patients showed no significant differences to those treated with LT4 in either evaluated scenario. However, noteworthy variations in TSH levels (*p* = 0.009) and ferritin (*p* = 0.031) were observed between euthyroid and LT4-treated patients in the ward, along with TSH (*p* = 0.044) and FT4 (*p* = 0.012) in the ICU. Moreover, parameters indicating an unfavorable prognosis were related to low FT3 levels and/or the age of the patient [139].

The relationship between COVID-19 outcome and thyroid hormone levels in infected patients has also been reported by Baldelli et al., who evaluated a cohort composed of three groups: (i) group A—COVID-19 patients; (ii) group B—COVID-19 patients in the ICU; and (iii) group C—control group [141]. Patients in groups A and B showed a significant reduction in FT3 and TSH compared with the control. Furthermore, group B exhibited a lower level of these hormones than group A, indicating that a hormonal imbalance occurs in patients with more severe conditions. The study by Lui et al. showed a decreasing trend of FT3 level with increasing severity of COVID-19, suggesting that patients with low levels of this hormone may experience severe adverse COVID-19 effects [142].

Despite scientific evidence about the coronaviruses harming the thyroid glands—as well as hormonal dysregulation in severe adverse health conditions—the studies observed in this topic focused on specific hormones, some of them employing classical techniques of clinical analysis. However, there are few studies exploring separation techniques and high-resolution mass spectrometry for the comprehensive analysis of metabolites in biological samples, especially considering that the thyroid glands play a significant role in metabolism regulation and are directly affected during SARS-CoV-2 infection. According to the previously selected cohorts, multivariate analysis clusters that discriminate the different conditions should have been evaluated. Thus, metabolomics approaches could be used to analyze and quantify these hormones, as well as broadly explore the metabolites in the infected organism associated with different organs.

### 3.8. Respiratory Diseases

Respiratory diseases affect the respiratory system, impairing the proper functioning of lungs, airways, and respiratory muscles [143]. COVID-19 predominantly impacts the respiratory system and can advance into a life-threatening systemic disease characterized by organ failure [144]. Studies published during the pandemic revealed that patients with respiratory diseases were at a higher risk of developing severe issues [145,146]. Common concurrent respiratory conditions encompass asthma, chronic obstructive pulmonary disease, pulmonary hypertension, lung cancer, pneumonia, tuberculosis, cystic fibrosis, sarcoidosis, extrinsic allergic alveolitis, and others [145,146,147,148,149].

Severe symptoms of COVID-19 were observed in patients with bronchial asthma at an age range from 1 month to 18 years old [150]. A higher prevalence of asthma among COVID-19-infected patients has also been observed in a single research center [151]. Despite these findings alerting to the risks of asthmatic patients developing severe COVID-19 conditions, other studies present divergent outcomes on the asthma–COVID-19 relation [152,153]. One possible explanation for this disparity could be related to different asthma endotypes [154]. Another hypothesis is that asthmatic patients are under medical supervision and, therefore, their behavior might be more cautious regarding viral infections [154]. Moreover, anti-inflammatory drugs such as corticosteroids administered to asthma patients may have a protective effect, although this is still unproven [154]. Nonetheless, the association between asthma and SARS-CoV-2 is considered complex, and further research is required to understand this divergence.

Luteolin, a medicinal compound, has exhibited antiviral and anti-inflammatory capabilities [155,156]. It specifically binds to the spike protein on the surface of SARS-CoV-2, hindering viral entry [157]. Additionally, Luteolin can inhibit the cytokine storm induced by IL-1β and histamine production in mast cells activated by SARS-CoV-2 [158,159]. Notably, it is a bronchoconstriction and airway hyperreactivity attenuator, holding promise as a therapeutic intervention for asthma [160]. The molecular mechanisms of luteolin addressing the COVID-19–asthma comorbidity suggest potential effects on virus defense, inflammation regulation, cell growth, and immune responses. It may help in reducing oxidative stress and regulating blood circulation [149]. While luteolin shows potential for treating COVID-19–asthma comorbidity, rigorous verification via genomic, proteomic, and metabolomics approaches is still essential [149].

The Mycobacterium tuberculosis bacillus is the causal agent of tuberculosis, a contagious infectious disease primarily affecting the lungs; additionally, other organs and tissues can be affected, namely, brain, kidneys, spine, and lymph nodes, in a condition known as extrapulmonary tuberculosis [161]. Tuberculosis results in one of the highest rates of morbidity and mortality globally. According to a WHO report, in 2021, approximately 1.6 million people died from tuberculosis, making it the second leading infectious cause of death in the world, after COVID-19 [161,162]. During the COVID-19 pandemic, support services for tuberculosis patients were limited, many individuals were affected, and the number of undiagnosed and untreated cases of the disease may have been under-reported [162].

Tuberculosis leads to different pulmonary outcomes, varying from no impairment to severe dysfunction [163]. Recovered tuberculosis patients may exhibit cavitation, nodular infiltrates, or fibrosis, and over one third may develop permanent lung damage [163]. Tuberculosis patients infected with SARS-CoV-2 belong to a higher risk group [164]. Potential biomarkers to predict morbidity and mortality of COVID-19 in patients previously affected by tuberculosis were identified by a metabolomics approach [147]. For this purpose, blood samples from 155 adults infected with SARS-CoV-2, of whom 23 had a history of tuberculosis and 132 did not, were analyzed by FIA-MS/MS and LC-MS/MS. The study revealed that a significant majority of post-tuberculosis individuals exhibited severe SARS-CoV-2 infection, demanding intensive oxygen support, and had a mortality rate of 52.2% [147]. Betaine and branched-chain amino acids (aspartate, glycine, serine, threonine, leucine, isoleucine, and valine) were identified as potential biomarkers for severity and mortality prognosis, respectively, in COVID-19 patients who had been exposed to tuberculosis [147]. However, the effects of pharmacological agents used in tuberculosis patients’ treatment and the posology should have been considered, to achieve more adequate group homogeneity for metabolomics analyses.

Patients with severe acute respiratory syndrome, even after treatment and discharge, may experience the early onset of pulmonary fibrosis [165]. Additionally, older patients and those with severe symptoms during treatment are more likely to develop lung problems [165,166]. Ergo, evaluation of molecular pathways involved in pulmonary sequelae represents a potential approach in metabolomics studies. The metabolic profile of patients who developed pulmonary sequelae three months after discharge was evaluated in plasma samples from 103 individuals recovered from COVID-19, alongside 27 healthy donors without a history of COVID-19 [167]. The detected changes were associated with the severity of the disease, primarily affecting amino acid and glycerophospholipid metabolic pathways [167]. Moreover, elevated levels of triacylglycerols, phosphatidylcholines, prostaglandin E2, and arginine, coupled with decreased levels of betaine and adenosine, were correlated with pulmonary CO diffusion capacity and total lung capacity [167]. Including asymptomatic groups in future studies could complement the conclusions obtained by this study.

The primary clinical presentation of a COVID-19 infection is pneumonia, a typical acute respiratory infection affecting the alveoli and distal bronchioles in the lungs [168]. On the other hand, conventional pneumonia is often caused by bacterial pathogens [169]. COVID-19 pneumonia has distinct radiological characteristics from conventional pneumonia, and several contributions have been made to differentiate them [170,171]. Approaches using artificial intelligence have been extensively explored to extract visual features from computed tomography scans [172,173,174]. The inflammation induced by pneumonia impacts blood circulation, and detectable biochemical changes can occur in the blood for both pathogens [148].

**Figure 7:**
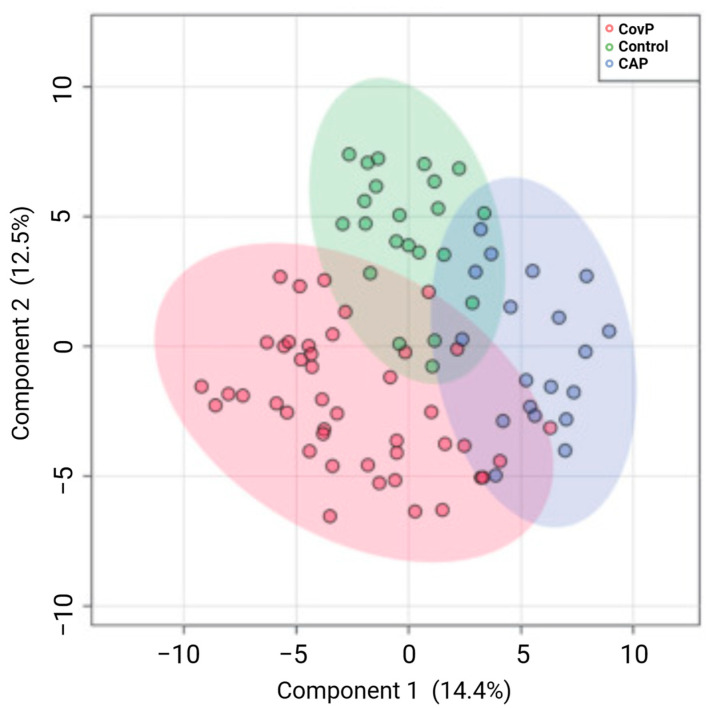
Score plot from principal component analysis illustrating the clustering of COVID-19 pneumonia (CovP) in red, control subjects (control) in green, and non-COVID-19 pneumonia (CAP) in blue. Link to the license, accessed on 14 March 2024 (https://creativecommons.org/licenses/by/4.0/deed.en). Image credit and adapted from More et al. [148].

A metabolomics study was conducted to compare the blood metabolome of patients with COVID-19 pneumonia and conventional pneumonia, divided into three groups: (i) 43 patients with COVID-19 pneumonia; (ii) 23 patients with conventional pneumonia; and (iii) 23 control individuals (without pneumonia). Sample analyses were performed by gas chromatography coupled to mass spectrometry (GC-MS). Metabolic shifts were noted within the three groups [148]. Principal component analysis (PCA) unveiled distinct clusters within the sample groups of COVID-19 pneumonia, non-COVID-19 pneumonia, and controls, as determined by their metabolic profiles (Figure 7) [148].

Elevated levels of aspartic acid, glycine, and serine were observed in the COVID-19 pneumonia group, whereas taurine levels were specifically increased in the COVID-19 pneumonia group and slightly decreased in the conventional pneumonia group [148]. Disease severity was correlated with the concentration of venous cytokines. Threonine was identified as a potential predictive biomarker for COVID-19 severity [148]. Nevertheless, the data analysis predictive model parameters exhibited relatively low values, probably due to the higher number of male participants compared with females, and different dietary factors and prescribed medication were disregarded, making the aforementioned results unreliable.

Unlike studies that rely on exhaustive and time-consuming sample preparation methods, a pilot study analyzed volatile organic compounds in the exhaled breath of COVID-19 patients experiencing acute respiratory distress syndrome and requiring mechanical ventilation [175]. Sample collection was conducted directly in the intensive care unit. The study involved 40 patients with acute respiratory distress syndrome, of whom 28 had confirmed COVID-19. A system was set up to collect samples directly from the breathing tube, and measurements were obtained using a proton transfer time-of-flight quadrupole mass spectrometer placed outside the patient’s room. The most notable volatile compounds observed in COVID-19 patients included methylpent-2-enal, 2,4-octadiene, 1-chloroheptane, and nonanal [175]. Another research using exhaled breath was conducted on COVID-19 patients (without the need for intubation) and healthy individuals [22]. Two-dimensional GC-MS was employed for untargeted metabolomic approach. The results indicated that the abundance of fatty acids could be used to discriminate COVID-19 patients [22]. Although the number of COVID-19 cases has fortunately been decreasing, both studies open possibilities for the development of rapid and less invasive tests in hospital settings for analyzing volatile compounds in patients with respiratory diseases.

Smoking is acknowledged as a risk factor for the development of various respiratory diseases, including lung cancer, asthma, and chronic obstructive pulmonary disease [176]. Thus, researchers have turned their attention to exploring the impact of smoking on the occurrence and progression of COVID-19 [177,178]. Upon assessing published works databases on this subject (smokers who contracted COVID-19), we built a database of proteomic and metabolomics studies [179]. Bioinformatics analysis was conducted to examine protein or metabolite interactions across the databases and their biological effects. The acquired data suggested that smoking might intensify the severity of COVID-19, and three metabolic pathways associated with immunity and inflammation, including tryptophan, arginine, and glycerophospholipid metabolism, were considered significant in the effect of smoking habits on adverse outcomes in COVID-19 patients [179]. The presented approach holds significant potential; however, the lack of standardization in metabolomics studies affects comparisons between different groups, since sample preparation methods and the use of different analysis platforms increase the variability of final data. According to the authors, more insights should be gleaned from existing studies to offer a more precise direction for future research, particularly in situations when conducting epidemiological studies is challenging [179].

### 3.9. Inducers of Comorbidities

#### 3.9.1. Malnutrition (Vitamins and Minerals)

Vitamins and minerals are regarded as the main regulators of human metabolism [180]. Thus, nutritional interventions are considered important in the immunization of the human population for disease prevention and as an adjuvant treatment for the relief of certain diseases [181]. Although the literature does not present a conclusive association between dietary supplementation and COVID-19 prevention, supplementation of some vitamins and minerals, such as vitamins C and D, zinc, and selenium has been recommended for elderly individuals and vulnerable groups [181,182,183]. A study performed in the beginning of the COVID-19 pandemic demonstrated that micronutrient (probiotics, omega-3 fatty acid, vitamins C and D, and zinc) supplementation in women significantly reduced the risk of SARS-CoV-2 infection [184].

Within the main micronutrient deficiencies, vitamin D is most associated with severity of COVID-19 [183,184,185,186]. However, 1 billion people worldwide are estimated to have below-recommended levels of this vitamin [187], which may be related to lifestyle and environmental factors that reduce exposure to sunlight, since natural production is induced from ultraviolet-B (UVB) radiation [188,189].

Low levels of vitamin D circulating in the organism result in high risk groups, including the following: (i) the elderly population, due to the fragility of the skin to synthesize the vitamin, more time spent indoors, and inadequate intakes [183]; (ii) dark-skinned people, due to increased melanin pigmentation, and the consequent reduction in vitamin D synthesis [190]; (iii) people with poor fat absorption, due to difficulty in solubilizing the vitamin [191]; (iv) obese individuals; and (v) people with limited exposure to sunlight. A hypothesis bolstering the significance of vitamin D in lowering the risk of COVID-19 suggests that the pandemic outbreak occurred during winter, a season associated with lower vitamin D concentrations in the body [186].

In addition to COVID-19, vitamin D deficiency is associated with worsening of chronic diseases such as cancer, heart disease, and T2D [187]. Vitamin D also showed an inverse association with body mass index (BMI) higher than 30 kg/m^2^, which also associates obesity with vitamin D deficiency [192].

The alveolar type II cells, also known as type II pneumocytes, have two functions: (i) repair of the alveolar epithelium when the sidewalk cells are injured, and (ii) secretion of pulmonary surfactant [193]. When the body is infected with SARS-CoV-2, alveolar dysfunction of type II pneumocytes occurs, thus increasing the risk of acute respiratory distress syndrome (ARDS) [194]. In this situation, vitamin D attenuates lung injury by decreasing apoptosis of epithelial cells and increasing surfactant synthesis [195].

According to a study conducted by Dror, A. A. et al. between April 2020 and February 2021, patients who tested positive for COVID-19 and who had a history of vitamin D deficiency were 14 times more likely to have severe disease symptoms than those with normal levels of the nutrient [185]. Among the individuals admitted to the medical center during this period, 253 had registers in the electronic medical record of vitamin D deficiency prior to COVID-19 infection [185].

Zinc, ranking as the second most abundant metal within the human body, plays a pivotal role in a multitude of cellular functions, notably contributing to the preservation of immune health [196]. Zn is associated with the regulation of immune cells, including natural killer (NK) cells, monocytes, neutrophils, and T and B lymphocytes. Zn may also increase the proliferation of CD8+ cytotoxic T lymphocytes, which are fundamental cells in the immune response against respiratory viruses [197]. It is also present in the functional maintenance of the barrier of the respiratory epithelium [198]. Copper also holds significance in the maintenance of the immune system. A lack of this metal is related to symptoms associated with copper-containing enzymes, such as superoxide dismutase, which serves to mitigate cell damage caused by oxidative effects [199].

A recently published study of 200 individuals (150 infected with COVID-19 and 50 healthy ones) showed the importance of Zn and Cu levels in the organism. The infected group was divided by COVID-19 severity (mild, moderate, and severe) and outcome (discharge or death). A direct colorimetric method was employed to analyze the levels of Zn, Mg, and Cu in the serum. Compared with the control group, the case group exhibited significantly lower Cu and Zn serum levels (*p* < 0.005 and *p* < 0.0001, respectively). Mg concentration showed no significant difference. The outcomes of the receiver operating characteristic (ROC) curve revealed that a serum Cu/Zn ratio, combined with patient age, furnishes dependable insights into the progression of COVID-19 and the likelihood of survival. The area under the curve (AUC) reached 95.1%, demonstrating a 93.8% sensitivity and 89.8% specificity [198].

A retrospective study in North Carolina compared the levels of selenium and zinc with the severity of COVID-19 among diagnosed individuals. To assess severity during the period of SARS-CoV-2 infection, individuals were asked about the existence and duration of 10 frequently reported symptoms. Toenail samples were analyzed to determine Se and Zn concentrations by ICP-MS/MS. Regression analysis was employed to evaluate the correlation between selenium (Se) and zinc (Zn) status and the severity of COVID-19. In this case, only selenium concentration showed an inverse association with COVID-19 severity (95% CI: *p* = 0.02) [200].

Individuals who received oral Zn supplementation have shown a 7-fold reduction of COVID-19 contamination compared with those who did not receive such treatment [201]. On the other hand, contradictory information showed that Zn, Se, and Cu concentration has no impact on SARS-CoV-2 infection, its severity, or hospitalization due to COVID-19 [202].

Despite numerous advances in research on vaccines, antibody treatments, and antiviral drugs for COVID-19, gaps regarding the importance of an individual’s nutritional status on the severity of COVID-19 are still present. However, readers who want to know more about antiviral properties of different vitamins and minerals, as well as the correlation between them and their effect on patients with COVID-19, may seek the following bibliographic references [203,204,205,206].

Micronutrients, including vitamins A, B, and C, as well as iron, manganese, selenium, zinc, and copper, have been reported to play a fundamental role in nutrition and human health. Despite the importance of each of them, studies present contradictions regarding the relevance of some nutrients in minimizing the effects of COVID-19, like vitamin C, and the minerals Zn, Se, and Cu [202,203,204,205,206,207]. Therefore, the effects of micronutrients on COVID-19 severity are still scarcely understood, since micronutrient deficiencies may be related to some diseases that alter the metabolic system of an individual. Thus, studies with other approaches may strengthen the functional comprehension of these micronutrients in the biological system.

#### 3.9.2. Immunological System

**Figure 8:**
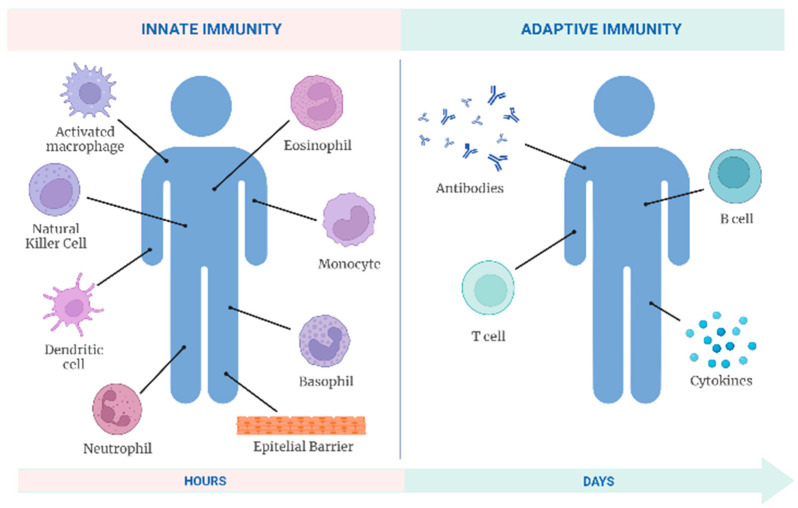
Cellular components involved in the innate and adaptive immune systems.

The immune system comprises a complex network of organs, cells, and proteins that play a critical role in protecting the human body against invaders, such as viruses, bacteria, fungi, and the related toxins [208]. It consists of two primary components: the innate immune system and the adaptive immune system. The former is inherent in individuals from birth and serves as the body’s initial defense against invaders, exhibiting a non-specific immune response. Conversely, the later performs specialized functions in antibody production and generation of memory cells, providing a more targeted and specific response (Figure 8) [209,210].

In the SARS-CoV-2 infection, as well as other microorganisms, the body initiates an inflammatory response triggered by vasodilation, which aims to increase blood flow to the affected region during injury or infection [209]. The dilatation of blood vessels facilitates the transport of white blood cells that release cytokines—chemicals involved in combating invaders. While inflammation plays a crucial role in eliminating the infection, excessive inflammation can lead to severe consequences [209,211].

A cytokine storm occurs when the innate immune system releases an excessive amount of cytokines simultaneously, resulting in systemic damage and potentially leading to multiple organ failure [211]. A study conducted during the pandemic revealed elevated cytokine levels in patients with severe COVID-19, supporting their association with disease severity [211]. To investigate the correlation between host metabolism and cytokine storm in COVID-19 patients, serum samples from four different groups were analyzed: (i) healthy controls (n = 17); (ii) mild COVID-19 patients (n = 14); (iii) severe COVID-19 patients (n = 23); and (iv) patients who tested negative for COVID-19 but exhibited respiratory tract infections with symptoms such as cough, fever, nasal congestion, among others (n = 20). These samples were prepared and analyzed by UHPLC-Q-TOF to conduct both targeted and untargeted metabolomics studies [211]. A total of 155 metabolites were annotated out of 6072 features. In terms of targeted metabolomics, 258 metabolites were monitored, and 134 of them were significantly detected as potential markers. Combining the two metabolomic approaches, 36 metabolites exhibited significant changes according to disease severity. Among the metabolites identified in severe cases using both metabolomics approaches, many of them demonstrated positive or negative correlations with the release of inflammatory cytokines, such as those related to arginine metabolism, namely arginine, glutamine, aspartic acid, citrulline, urea, and proline [211].

Metabolomics has also been applied to investigate the relationship between COVID-19 severity and pre-existing autoimmune diseases, such as the human immunodeficiency virus (HIV) [212]. Targeted metabolomics analysis revealed an increased ratio of phenylalanine to tyrosine in patients infected with COVID-19 and HIV. This finding raises the hypothesis that this alteration is associated with a deficiency of tetrahydrobiopterin (BH4), an important cofactor involved in metabolic pathways, including the conversion between phenylalanine to tyrosine [212]. Moreover, Ansone et al. revealed a distinct metabolomic profile in the serum samples of 32 hospitalized patients, highlighting differences between the acute and recovery phases. This implies a potential connection between tryptophan and arginine metabolism and the clinical severity of the disease, given their role in the immune response to SARS-CoV-2 [213].

Considering the aforementioned information, the obtained results underscore the significance of comprehending the intricacies of the immune system and the inflammatory reaction evoked by pathogens such as SARS-CoV-2. Consequently, the interplay between the innate and adaptive immune systems can furnish valuable perspectives for devising efficacious therapeutic strategies targeting COVID-19 and other infectious ailments. Nonetheless, additional metabolomic investigations are imperative to grasp the impact of autoimmune diseases on COVID-19 and how this inflammatory predisposition affects its severity.

#### 3.9.3. Oxidative Stress

Studies have indicated that despite the positive impact of COVID-19 pandemic on the environment, including the reduction in pollutants and subsequent improvement in air quality, it has had negative effects on the health and well-being of the population [214]. Bakadia et al. examined the consequences of stress induced by both biotic and abiotic factors on the onset of the pandemic, as well as catastrophic events such as floods, earthquakes, volcanic eruptions, and everyday situations like traffic congestion, work or relationship conflicts, noise, and even warfare. These stressors can lead to metabolic changes initiated by the brain, which can subsequently affect the immune system, resulting in an increased release of cytokines. Furthermore, cytokines stimulate oxidative stress by activating the reduced nicotinamide adenine dinucleotide phosphate (NADPH) [214].

**Figure 9:**
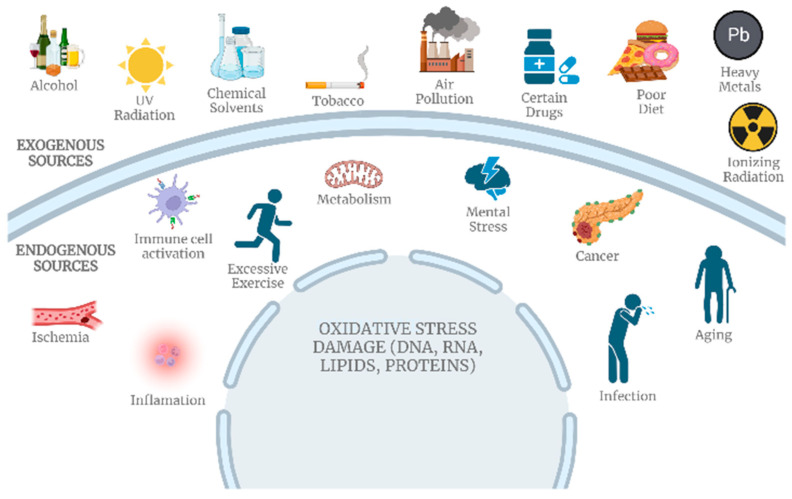
Sources of oxygen and nitrogen oxidative species.

Oxidative stress occurs when reactive species like reactive oxygen species (ROS) and reactive nitrogen species (RNS) are in disarray with antioxidants, ultimately resulting in tissue damage [215]. Both ROS and RNS can originate from external or internal sources (Figure 9) and are implicated in various diseases as triggers or contributors to disease complications. Additionally, metabolic reactions associated with oxidative stress play a significant role in initiating apoptosis, which is a potential mechanism associated with the reduction of T cells during COVID-19 [214].

Few metabolomics studies associate these stress factors with the development and severity of COVID-19, including the alteration of the white matter of the brain with the mental health and metabolism of patients recovered from COVID-19. Yang et al. investigated 28 recovered patients and 27 healthy subjects in the control group, with similar demographic characteristics [216]. Participants’ mental status was assessed by a psychiatrist using questionnaires and the Hamilton Anxiety Scale (HAMA) [217]. In addition, blood serum samples were collected for untargeted metabolomics analysis using UHPLC coupled to a Q Exactive high-resolution mass spectrometry (HRM) system; 22 metabolites showed significant increase in the case group compared with the control, whereas 30 metabolites showed significant reduction. Metabolites’ annotation resulted in xanthosine, adenosine, inosine, serotonin, and others, which impact on the patients’ mental health via the amino acid, lipid, and purine metabolic pathways, respectively [216]. The study revealed that all identified metabolites are associated with the inflammatory response of the body and play a crucial role in oxidative stress by promoting NADPH production [218]. This induction is facilitated by the increased inflammation, which is in turn related to the decrease in amino acid derivatives involved in the regulation of antioxidant species [219]. For instance, the enzyme cystathionine gamma lyase, responsible for converting cystathionine to cysteine, acts protectively against oxidative stress and is associated with reduced levels of glutathione in critically ill patients. Another significant compound is serotonin, and its depletion can lead to decreased blood oxygen levels [218].

Serotonin functions as a pulmonary vasoconstrictor and calcium-dependent activator of RNS in pulmonary endothelial cells, impacting the permeability of the endothelial barrier. Low levels of serotonin are strongly correlated with the release of Interleukin-7 (IL-7), which is exclusively expressed in thrombocytes that also store significant amounts of serotonin [218]. The reduced serotonin levels may be associated with decreased thrombocyte levels, explaining the development of thrombi in critically ill COVID-19 patients.

These findings underscore the significance of the interplay between pro-inflammatory cytokines, oxidative stress, and other metabolic components in the context of COVID-19. Gaining insights into these mechanisms is crucial for identifying potential therapeutic targets and developing effective intervention strategies aimed at mitigating oxidative stress, modulating the inflammatory response, and preventing severe complications associated with SARS-CoV-2 infection. Therefore, conducting further metabolomics studies is imperative to acquire a thorough comprehension about the intricate relationship between external and internal stressors, the severity of COVID-19 infection, and their implications for the overall health and mental well-being of the population, both during the ongoing pandemic and in the post-COVID period we have been currently experiencing.

## 4. Conclusions

After conducting a survey and searching databases on metabolomics studies involving COVID-19 and its comorbidities, we observed aspects that warrant reflection on the limitations and potentialities related to this subject. Obtaining biological samples from groups of patients with homogeneous clinical conditions is considered a challenge, and acquiring such samples may take several years in conventional metabolomics studies. In the face of the conditions imposed by the pandemic, this challenge has escalated concerning the planning and selection of groups for research. The urgency to report the outcomes of patients with pre-existing conditions to the scientific community took precedence over understanding the involved metabolic processes. Furthermore, logistical, safety, and ethical considerations may have influenced the availability of data and the procurement of samples for analysis.

A central observation in the evaluated studies is the presence of two or more comorbidities in patients with COVID-19. Cases like these are highlighted, for example, in patients simultaneously experiencing diabetes and obesity. Moreover, a number of comorbidities share overlapping metabolic pathways, such as kynurenine metabolism (observed in kidney disease, obesity, cancer, and in the immune system), fatty acid metabolism (noted in cardiovascular diseases, obesity, respiratory diseases, and diabetes), and amino acid metabolism (seen in diabetes, cancer, the immune system injury, and tuberculosis). This highlights the intricate interplay between various pathological conditions and metabolic processes. This observation underscores the focus on the intricate metabolic interactions between COVID-19 and the coexisting diseases, as well as the challenge faced by researchers in distinguishing the isolated effects of each comorbidity on the metabolic response associated with the virus.

The body of work related to observational studies of COVID-19 cases available in databases is considerable. Often, infected patients with comorbidities have been categorized into three groups based on symptom severity (mild, moderate, or severe). These observations have been highly significant throughout the course of the pandemic, since they assisted physicians in making decisions based on the presence or absence of pre-existing conditions or on patients’ daily habits, such as smoking or medication usage. However, studies that have considered the application of metabolomics are notably scarce. As a result, robust conclusions and the observation of consistent metabolic patterns across populations with different comorbidities are limited.

In light of this, the metabolic explanation that considers the interaction between COVID-19 and its comorbidities has gaps. Despite the considerable reduction in cases, strategies for studying this subject are still necessary to comprehend the underlying metabolic processes and pathways of interaction. One potential avenue for investigation is related to the sequelae of COVID-19. Research in this aspect could provide valuable insights into the long-term effects of the disease, as well as the metabolic association with specific comorbidities.

A detailed review of records of patient who have been infected with COVID-19 represents a potential guide for the selection of individuals who have exhibited severe conditions of the disease. Such an approach enhances the unveiling of metabolic patterns and contributes to a specific comprehension of metabolic implications in patients who experienced the most severe form of the illness. Additionally, the influence of different types of vaccines on groups with comorbidities deserves attention, since the metabolic profile and immune response may exhibit distinct outcomes. In light of the findings presented in this review, acknowledging the workflow in metabolomics research and the necessity for multidisciplinary effort to comprehend the complex metabolic interactions involved between COVID-19 and its comorbidities is crucial.

## Data Availability

No new data were created in this review.

## Supplementary Materials

The following supporting information can be downloaded at: https://www.mdpi.com/article/10.3390/metabo14040195/s1, Table S1: Summary of Metabolites Found upon bibliographic revision.
