# Supplementary material for: COVID-19 and Comorbidities: What Has Been Unveiled by Metabolomics?
Source: Metabolites. 2024 Mar 30;14(4):195. doi: 10.3390/metabo14040195 (PMC11051775; doi:10.3390/metabo14040195)
Supplement: Supplementary file 1 [file metabolites-14-00195-s001.zip › metabolites-2855844-supplementary.pdf]

Table S1: Summary of Metabolites Found upon bibliographic revision

Comorbidities	Metabolite	Biological matrix	Technique of analysis	Metabolomic approach	Altered metabolic pathways	References
Diabetes	Palmitic acid (C16:0), Docosapentaenoic acid (C22:5, DPA), Docosahexaenoic acid (C22:6, DHA).	Serum	FIA-MS/MS	Targeted	Triacylglycerols and long-chain polyunsaturated fatty acids (n3 and n6).	[48]
	Phenylalanine, Alanine, Citrulline, Proline.	Serum	FIA-MS/MS	Targeted	Phenylalanine metabolism, tyrosine metabolism, Arginine and citrulline metabolic pathways.	[49]
	Leucine, Isoleucine, Valine, Glutamate, Phenylalanine, Glutamine, Alanine, Proline, Cysteine	Plasma	UHPLC-MS/MS	Targeted	Amino-acid metabolism	[50]
	Sarcosine, Cholesterol esters, Aconitic acid, Spermidine, Glycosylceramides.	Serum	LC-MS/MS	Targeted	Glycosylceramide, Amino-acid metabolism, Cholesterol esters	[53]
	1,5-anhydro-D-glucitol (1,5-AG)	Serum	UHPLC-MS/MS	Targeted	Glucose	[54]
Obesity	Ceramides C18:0, C16:0, and C24:0; Tryptophan; Kynurenine; 3OH-Kynurenine.	Plasma	LC-MS/MS	Targeted	Tryptophan metabolism via kynurenine pathway and ceramide metabolism	[19]
	N6-Acetyl-L-Lysine, p-Cresol, mevalonic acid, phenol, 3,4-dihydrocymandelic acid, gallic acid, homo-l-arginine.	Plasma	UHPLC-MS/MS	Untargeted	homocysteine degradation, methyl histidine metabolism, catecholamine biosynthesis urea cycle, and glutathione-arginine-proline metabolisms.	[72]
Cancer	N1-acetylspermidine, N1,N8-diacetylspermidine, N1,N12-diacetylspermine, Indole-3-proprionate, 3-phenylproprionate.	Serum	UHPLC-QqQ and GC-QqQ	Untargeted and Targeted	Kynurenine metabolism, metabolism of acetylated polyamines.	[74]
	3-hydroxybutyrate, Lactate, Leucine, Phenylalanine, Cytokines.	Plasma	^{1}H-NMR	Untargeted	Glycolysis/gluconeogenesis; glyoxylate and dicarboxylate metabolism; glycine, serine and threonine metabolism.	[79]

Kidney disease	Lysine, Threonine, Leucine, Isoleucine, Phenylalanine, Tryptophan, Indoleacetic acid, hydroxyphenylpyruvic acid.	Urine	LC-MS	Targeted	Kynurerine–quinolinate pathway	[96]
Cardiovascular Diseases and Blood Disorders	L-arginine, 3-hydroxyoctanoylcarnitine, Creatine, 3,5-tetradecadiencarnitine	Plasma	UHPLC-MS	Untargeted	Coagulation cascade, platelet aggregation, myeloid leukocyte activation pathway, and arginine metabolism	[103]
	Ornithine, N-acetylornithine, 3-amino-2-piperidone, Aspartic acid, Asparagine.	Serum	UHPLC-MS	Untargeted	Ornithine, or urea cycle, correlation with multiple cytokine and coagulation indexes using metadata (*e.g.* active part thromboplastin ratio, international normalized ratio and prothrombin time)	[104]
	N-acetylneuraminate	Serum of SARS-CoV-2 infected hamsters	UHPLC-MS	Untargeted	Protein kinase signaling pathway	[105]
Thyroid disorders	Thyroid-stimulating hormone, Ferritin, Free thyroxine.	Serum	^{1}H-RMN	Targeted	Thyroid-stimulating Metabolism	[139]
Pulmonary tuberculosis	Aspartate, glycine, serine, threonine, leucine, isoleucine, valine, Betaine.	Serum	FIA-MS/MS and LC-MS/MS	Targeted	branched-chain amino acids (BCAAs), bile acid metabolism.	[147]
	2,4-octadiene, 1-chloroheptane, Nonanal	Expired air	GC-MS/MS	Untargeted		
Tobacco smoking	Tryptophan, arginine and glycerophospholipid	Serum	UHPLC-MS/MS and GC-MS/MS	Untargeted	Metabolic pathways related to immunity and inflammation	[179]
Immunological System	Succinate, Nicotinamide mononucleotide, Tryptofan, Kynurenine, Inosine,	Serum	UHPLC-MS/MS	Targeted	TCA cycle, NAD+ metabolism, tryptophan-kynurenine pathway, purine metabolism, phenylalanine metabolism.	[211]
	Phenylalanine, Tyrosine	Serum	^{1}H-RMN	Targeted		[212]
Oxidative stress	Inosine, Guanosine, Adenosine, Serotonin, Taurine.	Plasma	UHPLC-MS	Untargeted	Purine metabolism, tryptophan metabolism, taurine metabolism, glutathione metabolism.	[216]
	Glutathione	Plasma	LC-MS	Targeted		[218]
